# TP-Transfiner: high-quality segmentation network for tea pest

**DOI:** 10.3389/fpls.2024.1411689

**Published:** 2024-08-13

**Authors:** Ruizhao Wu, Feng He, Ziyang Rong, Zhixue Liang, Wenxing Xu, Fuchuan Ni, Wenyong Dong

**Affiliations:** ^1^ College of Informatics, Huazhong Agricultural University, Wuhan, China; ^2^ Engineering Research Center of Intelligent Technology for Agriculture, Ministry of Education, College of Informatics, Huazhong Agricultural University, Wuhan, China; ^3^ School of Computer Science, Wuhan University, Wuhan, China; ^4^ College of Plant Science & Technology, Huazhong Agricultural University, Wuhan, China

**Keywords:** tea pest, instance segmentation, dense and mimicry scenarios, attention mechanism, Mask Transfiner

## Abstract

Detecting and controlling tea pests promptly are crucial for safeguarding tea production quality. Due to the insufficient feature extraction ability of traditional CNN-based methods, they face challenges such as inaccuracy and inefficiency of detecting pests in dense and mimicry scenarios. This study proposes an end-to-end tea pest detection and segmentation framework, TeaPest-Transfiner (TP-Transfiner), based on Mask Transfiner to address the challenge of detecting and segmenting pests in mimicry and dense scenarios. In order to improve the feature extraction inability and weak accuracy of traditional convolution modules, this study proposes three strategies. Firstly, a deformable attention block is integrated into the model, which consists of deformable convolution and self-attention using the key content only term. Secondly, the FPN architecture in the backbone network is improved with a more effective feature-aligned pyramid network (FaPN). Lastly, focal loss is employed to balance positive and negative samples during the training period, and parameters are adapted to the dataset distribution. Furthermore, to address the lack of tea pest images, a dataset called TeaPestDataset is constructed, which contains 1,752 images and 29 species of tea pests. Experimental results on the TeaPestDataset show that the proposed TP-Transfiner model achieves state-of-the-art performance compared with other models, attaining a detection precision (AP50) of 87.211% and segmentation performance of 87.381%. Notably, the model shows a significant improvement in segmentation average precision (mAP) by 9.4% and a reduction in model size by 30% compared to the state-of-the-art CNN-based model Mask R-CNN. Simultaneously, TP-Transfiner’s lightweight module fusion maintains fast inference speeds and a compact model size, demonstrating practical potential for pest control in tea gardens, especially in dense and mimicry scenarios.

## 1 Introduction

As a vital economic crop, tea faces annual challenges from various pests during its cultivation, significantly impacting productivity and quality. Major tea pests include *Jacobiasca formosana*, *Geisha distinctissima*, *Arctornis alba*, *Measuring worm*, *Tortricida*, *Amata germana*, and *Euricania ocellus*, among others. Throughout the evolution of some tea pests, their morphological characteristics often undergo significant changes (Ihsan-ul Haq et al., 2003), making it difficult to manually track pest dynamics. Additionally, the mimicry and dense distribution characteristics exhibited by some tea pests complicate their identification and localization. Consequently, these challenges have driven the development of artificial intelligence for pest monitoring.

Convolutional neural network (CNN) is a primary choice for image processing and is widely used in various fields of computer vision. Sharma et al. (2022); Yang et al. (2024), and Singh et al. (2022) conduct image recognition and classification tasks across different application fields by constructing CNNs with various architectures. These studies leverage the excellent feature extraction capabilities of CNN and demonstrate the superiority and robustness of their respective models through experiments.

As deep learning continues to revolutionize various domains, its application in plant monitoring has garnered significant attention, leading to innovative solutions and enhanced performance in plant disease and pest detection. Liu and Wang (2021) explore challenges in the practical application of deep learning for plant disease and pest detection. They propose potential solutions, presented research ideas to address these challenges, and offered insightful suggestions. Kaur et al. (2024) utilized the H-CSM model, which integrates support vector machine (SVM), convolutional neural network (CNN), and convolutional block attention module (CBAM) to detect and classify plant leaf diseases. Experimental results indicate a classification accuracy of 98.72%. Kang et al. (2023) introduce MCUNet, a corn leaf pest detection and segmentation model that outperforms mainstream neural networks. Furthermore, aiming to obtain a more lightweight model, Agarwal et al. (2023) propose a pest detection method utilizing the EfficientNetB3 model. Experimental results demonstrate the effectiveness in achieving high accuracy for classifying various pests in image datasets. Dai et al. (2023) introduce an improved YOLOv5m-based method for pest detection in plants. By integrating Swin-Transformer and Transformer mechanism, their approach improves the detection accuracy and efficiency. Besides this, Jiao et al. (2022); Tian et al. (2023), and Yang et al. (2023a) also utilized deep learning methods to detect and classify pests on various plants. In summary, these studies have predominantly relied on conventional detection methods for monitoring and has not performed segmentation of the detected pests or leaf diseases. By achieving high detection accuracy through the construction of pest datasets and model improvements, these studies effectively address challenges such as small targets, multiscale detection, and real-time requirements.

In contrast, the field of pest or leaf diseases monitoring in tea gardens remains relatively underexplored, with only a few studies focusing on tea pest monitoring (Wang et al., 2023; Yang et al., 2023b; Ye et al., 2024). These studies primarily concentrate on the detection of tea pests without further segmentation of individual pests. The complex distribution of pests in tea gardens, characterized by mimicry and dense populations, presents significant challenges for traditional pest detection models. As for tea pest monitoring, a previous work conducted by Zhou et al. (2021) uses automatic machine learning to classify each image in the TeaPestDataset. Xue et al. (2023); Yang et al. (2023b), and Lin et al. (2023) utilize the popular object detection model YOLO (Redmon et al., 2016) to detect tea plant diseases or pests. Hu et al. (2021) employ a discriminative pyramid network for semantic segmentation of tea geometrids in natural scenes. Experimental results demonstrate excellent performance in the semantic segmentation of tea geometrids. In contrast, this research treats each pest as an individual entity, achieving specific pest counts and improving edge processing capabilities by developing a deeper network for instance segmentation. Furthermore, this study not only accurately identifies both larva and adult tea geometrids but also encompasses the identification and processing of 27 other common pests in tea gardens. Moreover, Hu et al. (2024) employ hybrid architecture based on transformer to detect tea pests in complex backgrounds. However, previous researches on tea pest monitoring primarily focus on classification, detection, or semantic segmentation tasks, ignoring the importance of instance segmentation tasks for pest control. This study summarizes previous researches on tea pest detection and applies instance segmentation tasks to improve the effectiveness of tea pest control. Instance segmentation offers a promising solution to these issues by enabling pixel-wise parsing of pest images, thereby accurately predicting the position of each pest.

Additionally, in practical applications, traditional detection methods face significant limitations, particularly in scenarios involving target overlap and occlusion, leading to suboptimal detection performance. Moreover, precise pesticide application in tea gardens necessitates adjusting dosages based on pest size to balance effective pest control with environmental concerns. Various pests and disease pathogens exhibit different degrees of resistance to pesticides at various growth stages. Consequently, pesticides should ideally be applied during periods when pests are most susceptible. The results of segmentation tasks can provide detailed information on pest growth, development, and distribution, which is critical for precise pesticide application.

To address these limitations caused by detection models, recent studies committed to segmentation tasks have shown potential solutions. Classical two-stage segmentation models, such as Mask R-CNN (He et al., 2017), Mask Scoring R-CNN (Huang et al., 2019), HTC (Chen et al., 2019), and DCT-Mask (Shen et al., 2021) exhibit excellent segmentation performance. Besides this, one-stage models such as BCNet (Ke et al., 2021) and SOLO (Wang et al., 2021) also have superior performance and efficiency. However, these segmentation models may lack sensitivity to details and edge features, leading to unsatisfactory extraction results and aliasing. Mask Transfiner (Ke et al., 2022) incorporates Transformer architecture into the model to provide supervision and self-correction for regions erroneously predicted by Mask R-CNN. Built upon this innovative mechanism, the segmentation performance of the edge area is significantly optimized.

The attention mechanism is a crucial component in various algorithmic theories within the realm of computer vision. The integration of the attention module with the deep network enhances the network’s ability to better extract target features (Xu et al., 2021)—for instance, Wang et al. (2022) demonstrate the effectiveness of the attention module combined with D2Det in pest segmentation. Yang et al. (2023b) improve the YOLOv7-tiny model by utilizing deformable convolution and attention mechanism, achieving 93.23% accuracy on their self-made tea pest segmentation dataset. Additionally, Zhang and Huang (2022) design a novel attention mechanism to overcome challenges such as scale changes, complex backgrounds, and dense distribution in light trap images. Experimental results show that the model outperforms both classic detection models and lightweight detection models.

Besides this, the deformable convolutional network (DCN) (Dai et al., 2017) enhances feature extraction accuracy by employing deformable convolution kernels. A deep convolutional network combined with a deformable convolution structure is proposed by Cao et al. (2020) to overcome geometric transformations. Experiments have demonstrated that the framework, when fused with the DCN, effectively improves the accuracy as well as inference speed of object detection. Significant improvement has been observed in the trade-off between them.

In order to effectively solve the monitoring problems of tea pests in mimicry and dense scenarios, this study proposes a framework named TeaPest-Transfiner (TP-Transfiner) for tea pest detection and segmentation tasks using an enhanced Mask Transfiner framework. The main contributions are as follows:

- Provide a dataset including 1,752 tea pest images and corresponding annotated file, which can be used in the object detection and instance segmentation tasks.
- Fuse the attention mechanism into the backbone network and improve the FPN architecture of the Mask Transfiner to get a novel pest monitoring model TP-Transfiner.
- Implement experiments and demonstrate that while maintaining lightweight, TP-Transfiner outperforms classical models for tea pest detection and segmentation tasks, particularly in dense and mimicry scenarios.

## 2 Materials and methods

This section summarizes the datasets used in this study and the implementation details of the proposed TP-Transfiner model. Specifically, Section 2.1 discusses the collection, annotation, and data augmentation of the TeaPestDataset. Section 2.2 details the overall process and implementation of the TP-Transfiner model. Section 2.3 presents the evaluation metrics used in the experiments.

### 2.1 TeaPestDataset and data augmentation

To develop widely applicable pest detection and segmentation models, a carefully selected and labeled dataset is necessary. In this study, various types of pest images in diverse scenarios are collected and manually labeled, resulting in a total of 1,752 images. The original pictures in the dataset are primarily sourced through three methods. The first method involved images provided by agriculture and forestry-related laboratories and pictures pertaining to tea pest knowledge. The second method consisted of on-site shooting in tea gardens using mobile devices. The third source was from Internet search engines. Consequently, the collected scenes are mainly categorized into indoor (laboratory or specimen) and outdoor (natural environment of the tea garden) scenes. Specifically, there are 1,492 images of outdoor scenes and 260 images of indoor scenes. These images serve as the original dataset for tasks related to the localization and segmentation of pest instances.

**Figure 1:**
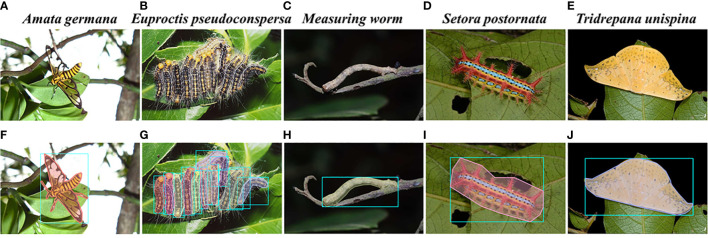
Original and annotated images of the pests. **(A–E)** Original and **(F–J)** ground truth.

**Figure 2:**
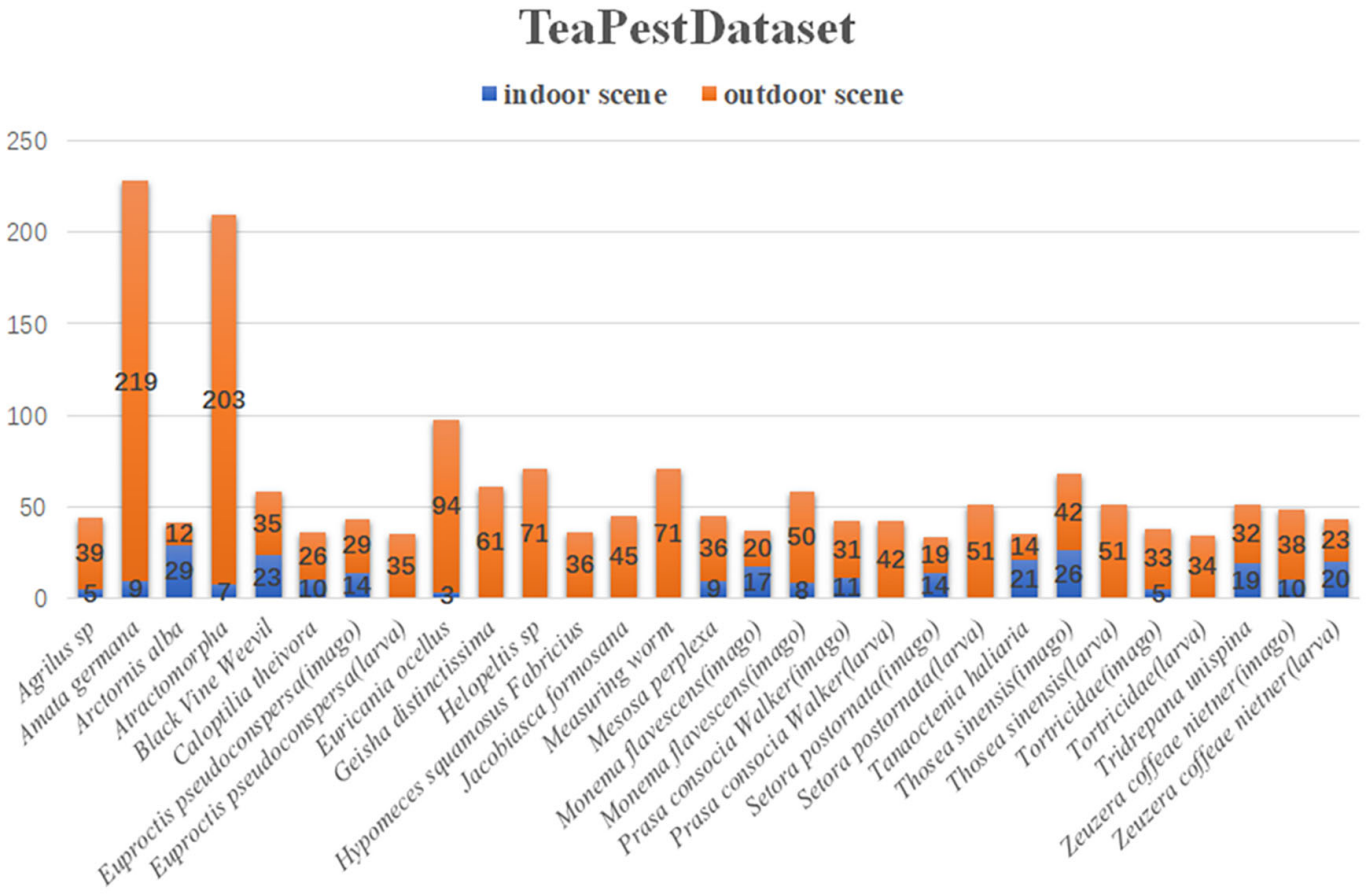
Schematic diagram of the number of types of data collection.

**Figure 1** presents samples of the dataset. The first row displays the original images, and the second row shows the annotated images. The dataset includes images and annotations of tea pests in mimicry and dense scenarios, providing a foundation for the model’s robust generalization performance in these complex scenes. During the dataset design process, 22 common pest species found in tea plantations are selected. However, considering the significant morphological differences during different growth stages of some pests, the larvae and adult stages of certain pests are further subdivided. Consequently, the final dataset comprises 29 categories, with the specific quantities of images in indoor and outdoor scenes for each type of pest illustrated in **Figure 2**.

**Figure 3:**
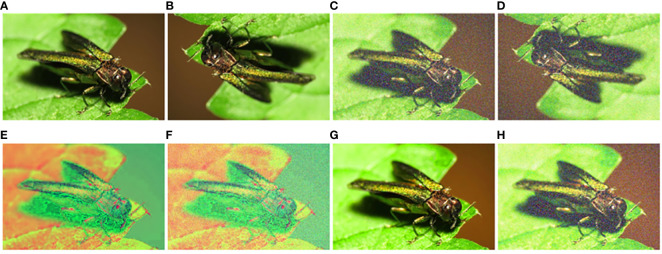
Examples of data augmentation. **(A)** Original, **(B)** flip, **(C)** noise adding, **(D)** flip and noise adding, **(E)** adjust hue 1, **(F)** adjust hue 2, **(G)** adjust saturation, and **(H)** adjust saturation and noise adding.

The initial sample size is limited, and there is inconsistency in the number of various data types. This condition may lead to model overfitting, causing a tendency to predict categories with a higher number of samples. Hence, the original dataset is augmented to achieve a more uniform distribution of each data type. In addition to rotation and cropping, random affine transformations and random color transformations (including adjustments to image brightness, contrast, saturation, and hue) are applied to enhance the model’s generalization ability, as shown in **Figure 3**. Finally, the dataset in this study includes a total of 34,928 images across 29 categories. The process of making the dataset is to divide the 1,752 original images in a ratio of 7:2:1 and then perform data augmentation on the training set and validation set. To avoid falsely high precision, the test set remains the original images.

### 2.2 TeaPest-Transfiner

**Figure 4:**
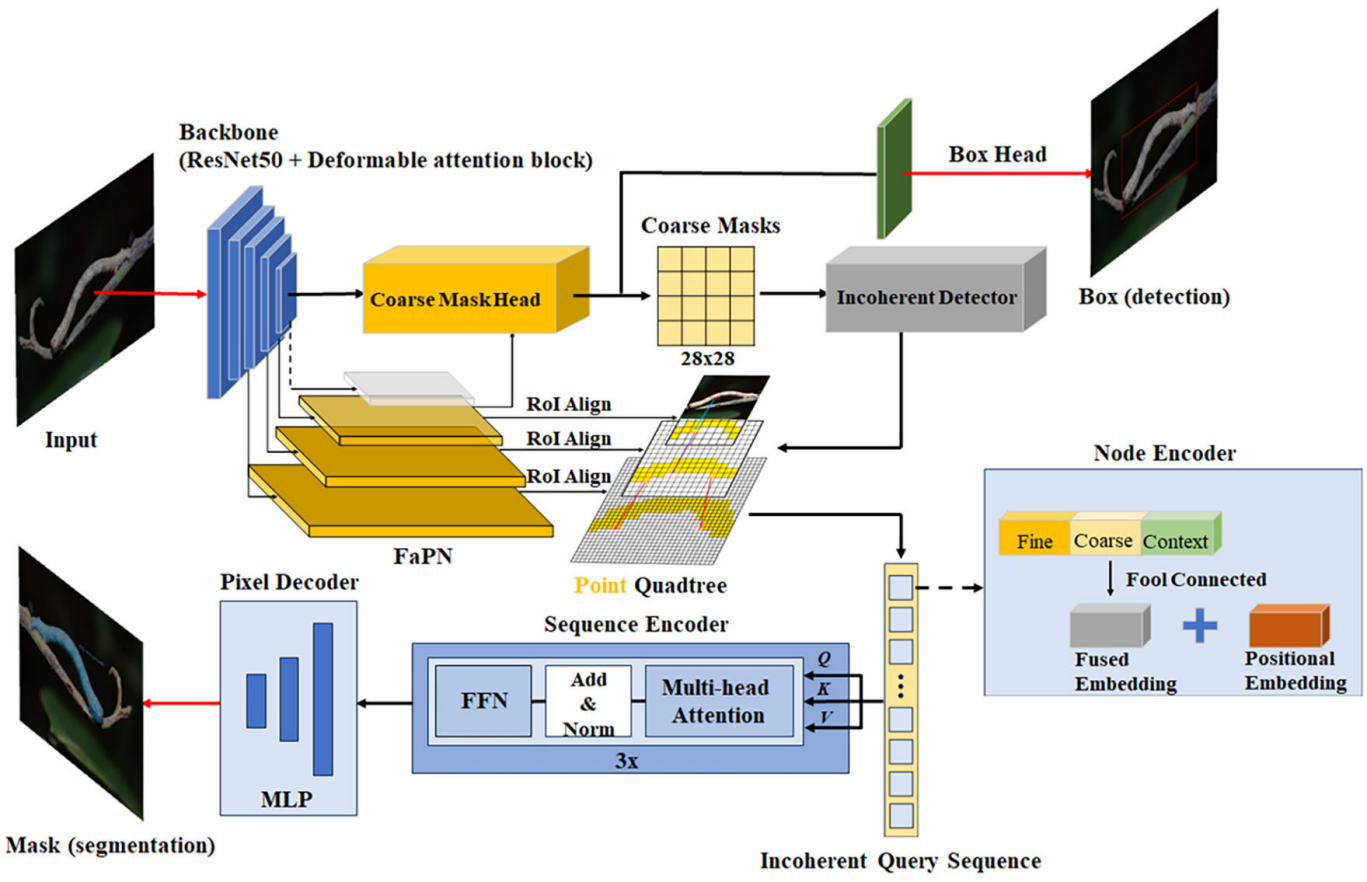
Framework of TP-Transfiner.

This study introduces an optimized framework—for instance, segmentation of tea pests based on Mask Transfiner. Primarily, it integrates the attention mechanism and DCN module into backbone network, replacing the backbone network in Mask Transfiner. Additionally, it utilizes the feature-aligned pyramid network (FaPN) (Huang et al., 2021) as a feature extraction module to segment the edge of each instance in high quality. **Figure 4** depicts the network diagram of the optimized Mask Transfiner segmentation model, referred to as TP-Transfiner.

#### 2.2.1 Backbone network

Most of the time, backbone network refers to the feature extraction network, and its function is to extract information from the image, which is then utilized by the box head and mask head. In this study, a ResNet fused with attention module and FaPN are combined as the backbone network of Mask Transfiner, which is used to extract features of pests.

##### 2.2.1.1 ResNet

**Figure 5:**
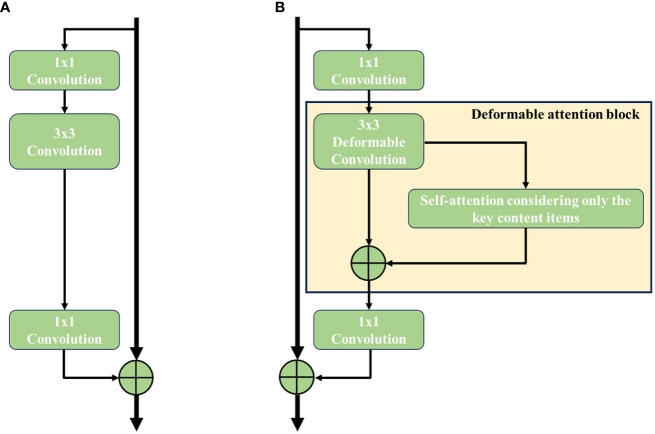
Structure comparison of residual block in ResNet-50. **(A)** Original residual block and **(B)** deformable attention block.


The ResNet is proposed by He et al. (2016), and it has been proven to effectively improve the accuracy and convergence of deep learning. A ResNet learns image data by its well-designed residual block (as shown in **Figure 5A**), which can be defined as Equations 1 and 2.

(1) $$y=F\left(x,\left\{W_{i}\right\}\right)+x$$

(2) $$y=F\left(x,\left\{W_{i}\right\}\right)+W_{s}x$$

where *F*(*x*, {*W_i_*}) denotes the residual mapping to be learned, and *x* is the input vector of previous layers or image. If the dimensions of *x* and *F* are not equal, a linear projection *W_s_* can be applied to match the dimensions, as shown in formula 2.

According to the research of He et al. (2016), the experimental results illustrate that the residual block has the ability in solving problems such as gradient vanishing and training degradation of the deep network. ResNet has outstanding feature extraction performance without increasing the model parameters and computational burden. Therefore, ResNet is chosen as the backbone network. At the same time, to balance efficiency and accuracy, ResNet-50 is chosen.

##### 2.2.1.2 Attention mechanism

The attention mechanism in deep learning draws inspiration from the attentional processes observed in human vision. Essentially, it comprises a set of weight parameters that can autonomously learn during the training period through the network. The mechanism prioritizes region of interest (RoI) in a dynamically weighted manner, simultaneously suppressing irrelevant background regions.

Dai et al. (2019) propose a solution to address the issue of context fragmentation by integrating the transformer attention module into the backbone network. Building upon this foundation, Zhu et al. (2019) conduct a comprehensive study that investigated the influence of four different factors: the query and key content, the query content and relative position, the key content only, and the relative position. Additionally, they explore the impact of incorporating deformable convolution into the network. Empirical results show that a proper combination of deformable convolution and the key content only term in transformer attention achieves the best accuracy–efficiency trade-off compared with the transformer attention module alone. Based on this conclusion, the key content self-attention module is integrated into the ResNet-50 backbone network in this study. Detailed information is indicated by Equation 3.


(3) $$\xi =u_{m}^{T}V_{m}^{C}x_{k}$$

where *u_m_* is a learnable vector. It captures salient key content which should be focused on the task and is irrelevant to the query. *T* represents the transpose of a vector, and *m* represents one of the attention heads. $V_{m}^{C}$ is learnable embedding matrices for the key content and *x_k_* denotes the input.

Specifically, the 3 × 3 regular convolution in the residual block is replaced with a deformable convolution block. Subsequently, a 3 × 3 deformable convolution in the residual block is followed by the addition of a self-attention module, contributing to the deformable attention block (as shown in **Figure 5B**). To apply a pre-trained model without altering its original behavior, the self-attention module is inserted using a residual connection. The output of the self-attention module is then multiplied by a learnable scalar initialized to zero. The residual block after the third stage of ResNet-50 is replaced with an optimized one, and the feature map outputted by ResNet-50 serves as the input for FaPN for multi-scale feature extraction.

##### 2.2.1.3 FaPN

**Figure 6:**
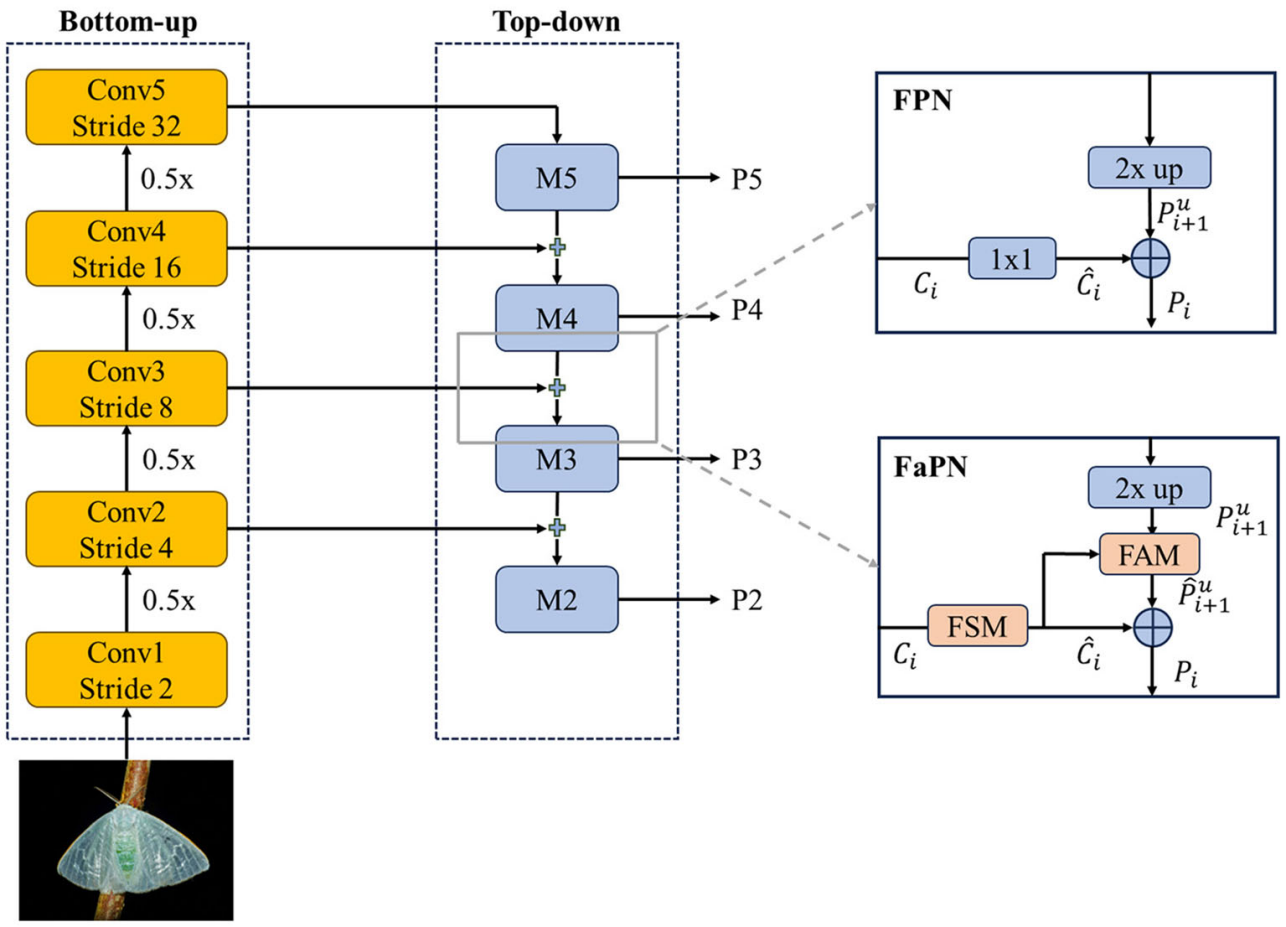
Structural comparison of FPN and FaPN.


Achieving accurate mimetic pest instance detection requires the availability of both high-quality spatial information for precise object detection and robust semantic information for effective classification. FaPN optimizes FPN by replacing 1 × 1 convolutions with a feature selection module (FSM) and adding a feature alignment module (FAM) during upsampling, as shown in **Figure 6**. Inspired by SENet (Hu et al., 2018), FSM accurately extracts crucial information about features and recalibrates them by performing channel reduction and suppressing redundant feature maps. FSM can be represented by Equation 4.

(4) $$\hat{C_{i}}=F_{s}\left(C_{i}+f_{m}\left(z\right)*C_{i}\right)$$

Here *z* signifies the data obtained through global average pooling of the input feature map *C_i_*, while *f_m_* (*z*) denotes the modeling of the importance of each feature map through a process involving a 1 × 1 convolution followed by a sigmoid activation on *z*.

FAM refines each sampling position within the convolution kernel by employing a learnable offset, thereby aligning the upsampled feature map with a set of feature maps. The feature map *C_i_*_-1_ furnishes the spatial position to determine *P_i_*, ensuring alignment with *C_i_*_-1_. FAM can be explained by Equation 5:


(5) $$\hat{P_{i}}\;=F_{a}\left(P_{i},f_{\circ }\;\;\left({\hat{C}}_{i-1}\circ \;P_{i}\right)\right)$$

where ∘ signifies the channel concatenation operation, *f*_°_ denotes the learned offset, and *F_a_* (·) represents the alignment function.

#### 2.2.2 Segmentation algorithm

To avoid a large number of edge pixels being misclassified, Mask Transfiner considers not only the high-level semantics of the image but also the large-resolution deep feature maps. With these fusion features, Mask Transfiner gains better result than the classic framework for tea pest detection and segmentation tasks in dense and mimicry scenarios. Besides this, the bounding box used for the detection task is generated by the original Faster R-CNN (Ren et al., 2016).

The mask head of Transfiner employs a quadtree structure to represent discrete points at various levels, addressing the discrete distribution characteristics of information loss areas. It utilizes a segmentation network based on Transformer to predict the label of each tree node instance in discontinuous space. As shown in **Figure 4**, the network comprises three modules—node encoder, sequence encoder, and pixel decoder—which work together to convert discrete nodes into unordered pixel sequences and predict instance labels for each point.

#### 2.2.3 Loss function

Based on the structures above, the entire Mask Transfiner framework can be trained in an end-to-end manner. As shown in Equation 6, a multi-task loss function is defined as:


(6) $$L=\lambda_{1}\;L_{Detect}+\lambda_{2}L_{Corase}+\lambda_{3}L_{Refine}+\lambda_{4}L_{Incoherent}$$

Here *L_Refine_* signifies refinement with L1 loss between predicted labels for incoherent nodes and their ground-truth labels. In TP-Transfiner, *L_Refine_* is replaced with smooth L1 loss. Besides this, a binary cross-entropy loss *L_Incoherent_* is utilized for detecting incoherent regions. The detection loss, denoted as *L_Detect_*, encompasses both localization and classification losses derived from the base detector, exemplified by Faster R-CNN. Subsequently, *L_Coarse_* represents the loss attributed to the initial coarse segmentation prediction generated by Mask R-CNN. The weights *λ*_1,2,3,4_ are initially given as {1.0,1.0,1.0,0.5}, respectively.

To mitigate the challenge posed by mimetic and close contact instances, focal loss (Lin et al., 2017) is introduced to *L*_Coarse_ during training. Focal loss is tailored to address class imbalance in object detection tasks, where background class pixels dominate. Traditional cross-entropy (CE) loss struggles with the surplus of background samples, hindering optimal learning for the minority foreground class. Similarly, the mimicry of tea pests requires TP-Transfiner model to pay more attention to instances camouflaged within the background during training.

##### 2.2.3.1 Focal loss

Yao et al. (2022) utilize focal loss to train Mask R-CNN and Mask Scoring R-CNN for peach disease segmentation. Experimental results indicated that after parameter adjustment, focal loss not only enhances segmentation accuracy but also improves detection rate. Based on this conclusion, focal loss is introduced to *L*_Coarse_ to enhance the performance of TP-Transfiner, and parameters are adjusted in the same way.

Focal loss introduces a modulating factor that down-weights the contribution of well-classified examples, focusing more on the hard-to-classify samples. The key idea is to assign lower weight to easily classified examples and higher weight to misclassified or challenging examples. Equation 7 shows detailed definition for focal loss.


(7) $$FL\left(p_{t}\right)=-\sum_{i=1}^{N}\alpha_{i}{\left(1-p_{i}\right)}^{\gamma }\text{log}\left(p_{i}\right)$$

Here *p_t_* represents the predicted probability of the true class, and *γ* is a focusing parameter initially defined. Notably, *α_i_* represents the category weight assigned to each sample, where samples belonging to the same category share identical weights.

### 2.3 Evaluation metric

This study primarily focuses on object detection and instance segmentation tasks. Mean average precision (mAP) serves as a commonly used evaluation metric in object detection. Araújo et al. (2019) and Hong et al. (2020) proposed that its corresponding index is the average of the average precision rate (mAP). This metric is calculated using the values of true positive (TP) and false positive (FP) to assess the detection and segmentation results. Equations 8 and 9) can be employed for calculation. The higher the two parameters are, the better the detection and segmentation results.


(8) $$Bbox-mAP=mean\left(\frac{TP}{TP+FP}\right)$$

(9) $$Seg-mAP=\sum_{i=1}^{k}\frac{AP\left(i\right)}{C}$$

where TP, FP, and FN represent true positive, false positive, and false negative, respectively. AP is the average precision of pixels segmentation, and *C* is the number of segmentation categories. Furthermore, AP50 and AP75 in detection task represent mAP of Bbox when IoU is 0.5 and 0.75, respectively. Also, AP50 and AP75 in segmentation task represent mAP of mask when IoU is 0.5 and 0.75, respectively.

## 3 Results and discussion

This section summarizes all the experiments and related extended discussions conducted in this study to demonstrate the effectiveness of the TP-Transfiner model. Section 3.1 presents the hyperparameter settings and the training process of the model. Section 3.2 discusses the results of adjusting two parameters in focal loss. Section 3.3 compares the TP-Transfiner with state-of-the-art models. Section 3.4 details the ablation study of the model.

### 3.1 Implementation

The experiments in this paper are conducted in Linux environment of the CentOS system, utilizing Python 3.7 and the PyTorch 1.7.1 framework. Two NVIDIA Tesla V100 32 GB GPUs are employed for training. Stochastic gradient descent (SGD) with momentum is chosen as the optimization method during training, with a momentum parameter set to 0.9 and 1K constant warm-up iterations. Besides this, the initial learning rate is set to 0.01, with a weight decay factor of 0.0001. The batch size is 8, and the training process extends over 12 epochs. The learning rate is reduced to 0.1 times the original value after the 8th and 11th epochs, respectively. After each epoch, the model is validated on the validation set and the weights of the current model are saved. The Mask Transfiner encoder consists of three standard transformer layers. Each layer has four attention heads with feature dimension at 256. Furthermore, the improved Mask Transfiner is initialized using the original Mask R-CNN model pre-trained on the COCO dataset (Lin et al., 2014) to accelerate the training process. All experiments are conducted on Detectron2 (Wu et al., 2019).

### 3.2 Adaption of parameters

In the current study, focal loss is utilized with empirical values of *γ* = 2 and *α* = 0.25. However, it is noted that different data distributions may require different parameters. Therefore, various values of *γ* and *α* are tested to accommodate these variations. As shown in **Table 1**, the implementation of focal loss enhances the overall accuracy of TP-Transfiner, with BCE loss resulting in the lowest accuracy. For each *γ*, the optimal *α* is determined to fit the dataset. As *α* increases, the weight of difficult samples increases, but excessively large *α* values can decrease the accuracy of the model. **Table 1** demonstrates that the experimental results align well with these observations. The table only displays detailed results when *γ* = 2. For *γ* = 1,3,4,5, only the optimal results are shown. After multiple rounds of testing, the model achieves the best result on the validation set when *γ* = 2 and *α* = 0.45. As a result, focal loss improves the overall segmentation accuracy by 2.1%.

**Table 1:** Training parameter and test results based on TP-Transfiner with different loss functions.

Model	Bbox_mAP (%)	Segm_mAP (%)	Loss type		Epoch	*γ*	*α*
			*L_Coarse_*	*L_Refine_*			
TP-Transfiner	67.499	64.000	BCE	Smooth L1 loss	12		
TP-Transfiner	68.501	65.650	Focal	Smooth L1 loss	12	2	0.25
TP-Transfiner	67.875	65.744	Focal	Smooth L1 loss	12	2	0.35
TP-Transfiner	67.372	**66.123 (+2.1)**	Focal	Smooth L1 loss	12	2	0.45
TP-Transfiner	68.247	65.913	Focal	Smooth L1 loss	12	2	0.55
TP-Transfiner	67.359	64.851	Focal	Smooth L1 loss	12	2	0.75
TP-Transfiner	67.259	63.473	Focal	Smooth L1 loss	12	2	0.95
TP-Transfiner	67.960	65.290 (+1.3)	Focal	Smooth L1 loss	12	1	0.45
TP-Transfiner	67.834	65.237 (+1.2)	Focal	Smooth L1 loss	12	3	0.55
TP-Transfiner	67.900	65.217 (+1.2)	Focal	Smooth L1 loss	12	4	0.55
TP-Transfiner	67.791	65.010 (+1.0)	Focal	Smooth L1 loss	12	5	0.45

The bold value indicates segmentation accuracy when the model performs best. The values ​​in brackets are the added values ​​compared to the first row of **Table 1**.

**Figure 7:**
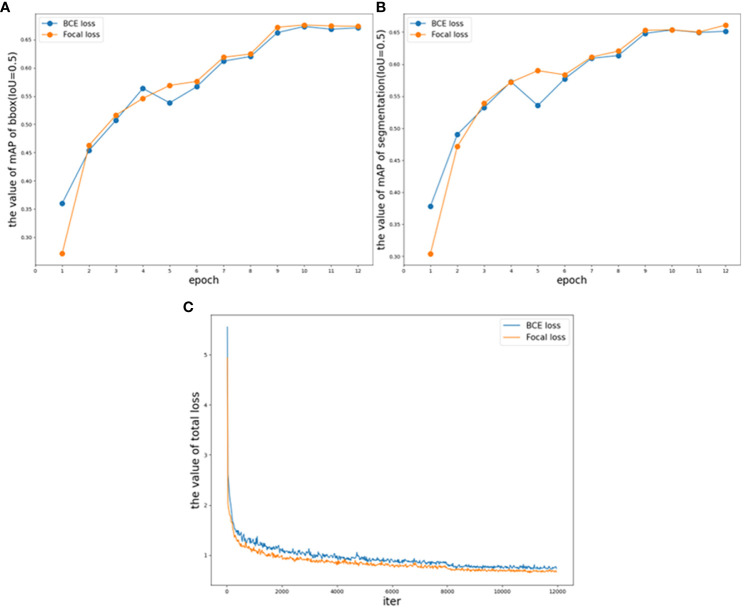
TP-Transfiner with different loss validation parameters and loss functions. **(A)** Comparison of mAP of bbox (IoU = 0.5) on different loss, **(B)** comparison of mAP of segmentation (IoU = 0.5) on different loss, and **(C)** comparison of total loss.

To illustrate the optimization achieved with focal loss, the accuracy on the validation set and changes in loss during the training period are depicted. **Figure 7A** presents the validation mAP of bounding boxes (IoU = 0.5) from epoch 1 to epoch 12 when training the dataset with different loss functions, indicating that the validation mAP of bounding boxes is higher with focal loss compared to BCE loss. **Figure 7B** shows the validation mAP of segmentation (IoU = 0.5) over the same epochs when trained with different loss functions, similarly demonstrating that the mAP of segmentation is higher with focal loss. **Figure 7C** illustrates the trend of training loss under different loss functions. It is evident that focal loss effectively reduces the loss during the training period compared to BCE loss, making the model more suitable for the distribution of the dataset. The results shown in **Figure 7** and **Table 1** indicate that the application of TP-Transfiner with focal loss achieves superior performance compared to BCE loss.

### 3.3 Comparison to state-of-the-art models

**Figure 8:**
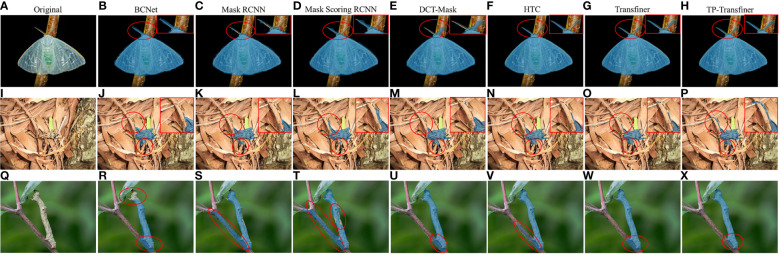
Comparison of segmentation results of different state-of-the-art models for tea pests with detailed and mimetic feature. **(A–H)** *Arctornis alba*, **(I–P)** *Mesosa perplexa*, and **(Q–X)** *Measuring worm*.

Detecting and segmenting pests in mimicry and dense scenarios poses a formidable challenge in tea production industry. The proposed TP-Transfiner model demonstrates excellent performance in addressing the detection and segmentation tasks especially in dense and mimicry scenarios. As illustrated in **Figures 8A–H**, conventional models such as BCNet, Mask R-CNN, Mask Scoring R-CNN, DCT-Mask, and HTC struggle to precisely segment intricate parts like antennae. Similarly, Mask Transfiner encounters difficulty in effectively capturing detailed features. In contrast, TP-Transfiner exhibits outstanding performance in accurately detecting and segmenting pests with detailed characteristics.

#### 3.3.1 Performance in mimicry scenarios

Some tea pests like *Measuring worm* and *Mesosa perplexa* are very good at using the surrounding environment to disguise themselves. This phenomenon, called mimicry, greatly increases the difficulty of the neural network to detect tea pests. Through superior edge feature extraction ability, TP-Transfiner demonstrates excellent performance in detecting and segmenting mimetic pests. In **Figures 8I–P**, though various models segment the pest camouflaged in leaves, TP-Transfiner distinguishes itself by segmenting the detailed antennae and small body. Besides this, as shown in **Figures 8Q–X**, BCNet, Mask R-CNN, Mask Scoring R-CNN, DCT-Mask, and HTC all misidentify branches as pests. The original Mask Transfiner slightly improved the situation, while TP-Transfiner improves the segmentation of the mimetic pest very well. As a result, the proposed TP-Transfiner can effectively detect and segment the specific contours of tea pests in such scenarios.

#### 3.3.2 Performance in dense scenarios

**Figure 9:**
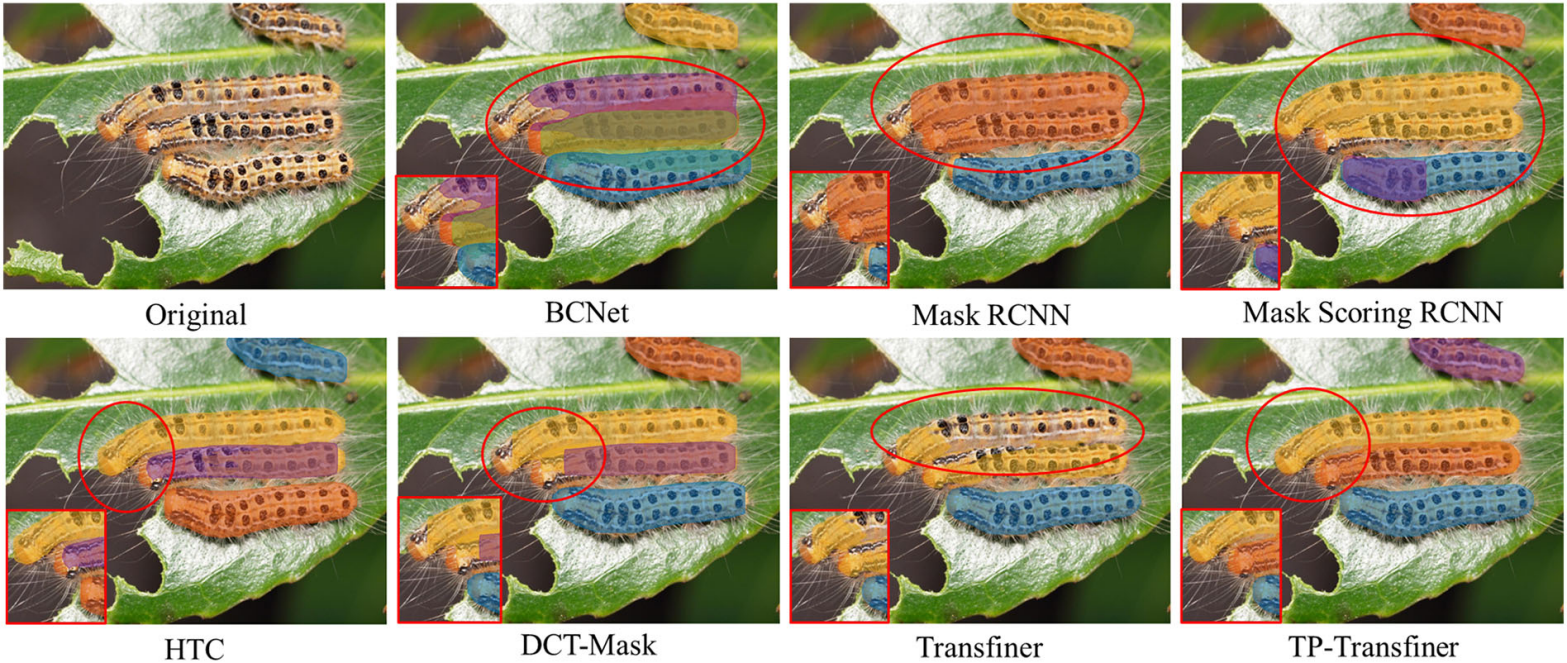
Comparison of segmentation results of different state-of-the-art models for tea pests (*Euproctis pseudoconspersa*) in dense scenarios.

In the dense scenario depicted in **Figure 9**, the segmentation results of TP-Transfiner significantly outperforms other models. Though some models fail to segment two instances in contact (BCNet, Mask R-CNN, and Mask Scoring R-CNN) or segment overlapping objects, TP-Transfiner performs well. Additionally, TP-Transfiner demonstrates powerful ability in detail processing. Compared to other models, the mask predicted by TP-Transfiner comprehensively covers the entire detected pests, while BCNet, Mask R-CNN, Mask Scoring R-CNN, HTC, DCT-Mask, and Mask Transfiner retains a large number of unpredicted pixels belonging to pests. Overall, TP-Transfiner demonstrates superior edge feature extraction ability compared with other models, enabling accurate detection and segmentation of tea pests in dense distribution.

Besides this, this study compares the detection and segmentation accuracy of seven state-of-the-art models including BCNet, Mask R-CNN, Mask Scoring R-CNN, HTC, DCT-Mask, Mask Transfiner, and TP-Transfiner. The results are shown in **Table 2**. Compared with BCNet, Mask R-CNN, Mask Scoring R-CNN, DCT-Mask, and HTC, the original Transfiner has obvious advantages in instance segmentation, and BCNet and HTC have higher accuracy in object detection task. Subsequently, the study optimizes the Transfiner by integrating deformable convolution, attention mechanism, and FaPN, resulting in the TP-Transfiner. Comparative analysis reveals that TP-Transfiner outperforms other methods, achieving the highest detection accuracy (mAP) of 67.372% and segmentation accuracy (mAP) of 66.123% for object detection and instance segmentation tasks. As for the light weight of the model, TP-Transfiner has a more significant advantage than other segmentation framework (except the original Transfiner). It denotes that TP-Transfiner holds broader application prospects in tea gardens with limited hardware equipment.

**Table 2:** Comparison to different state-of-the-art models.

Model	Model size (MB)	Detection			Segmentation		
		mAP (%)	AP50 (%)	AP75 (%)	mAP (%)	AP50 (%)	AP75 (%)
BCNet (one-stage)	292.90	59.330	76.910	67.151	52.498	75.301	58.357
MS R-CNN	460.20	56.705	84.605	64.578	56.547	81.881	63.909
Mask R-CNN	335.92	56.971	84.419	66.690	56.704	83.114	63.562
HTC	590.40	61.923	83.300	69.713	57.956	80.821	65.301
DCT-Mask	736.23	57.571	83.304	66.378	58.057	82.768	66.289
Transfiner	202.35	59.886	85.067	68.738	60.687	84.871	69.149
**TP-Transfiner**	235.07	**67.372**	**87.211**	**76.271**	**66.123**	**87.381**	**76.002**

The six bold values are the accuracies of the best models. For the specific meaning of accuracies, refer to the table header.

### 3.4 Ablation study

#### 3.4.1 Impact of deformable attention block

**Figure 10:**
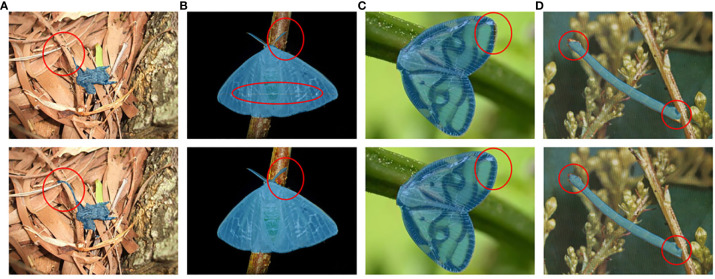
Comparison of segmentation results before (the first row) and after (the second row) integrating the deformable attention block. **(A)** *Mesosa perplexa*, **(B)** *Arctornis alba*, **(C)** *Euricania ocellus*, and **(D)** *Measuring worm*.

To evaluate the feature extraction ability of the deformable attention block on transparent wings, slender antennae, and legs of tea pests, detailed comparative experiments are conducted. **Figure 10** illustrates the segmentation effects of two different modules on pests with varying characteristics. It is evident that the model integrating the deformable attention block significantly improves the detection and segmentation effect of pest antennae (A, B), transparent wings (B, C), and mimicry scenario (D). The results demonstrate the deformable attention block’s exceptional feature extraction ability for pest’s edges and transparent states. The impact of the deformable attention block on detection and segmentation accuracy will be illustrated in the next section.

#### 3.4.2 Effect of different modules

**Table 3** illustrates that the integration of various modules into the Transfiner framework yields distinct accuracy improvements, with a more pronounced enhancement observed upon combining three modules. Compared with Transfiner, the proposed TP-Transfiner model improves the object detection accuracy (mAP) by 8.6% and the segmentation accuracy (mAP) by 5%. In addition, the fusion of modules does not affect the inference speed on images.

**Table 3:** Comparison of models after integrating different modules.

Attention	DCN	FaPN	Backbone	Runtime (FPS)	Detection			Segmentation		
					mAP (%)	AP50 (%)	AP75 (%)	mAP (%)	AP50 (%)	AP75 (%)
			ResNet-50	9.7	59.886	85.607	68.738	60.687	84.871	69.149
		**√**	ResNet50	7.7	64.915	86.167	74.938	63.811	85.744	73.222
**√**			ResNet50	9.1	61.434	85.937	71.586	61.498	84.761	70.802
**√**		**√**	ResNet50	7.3	64.416	86.237	75.089	63.539	85.989	72.271
	**√**		ResNet50	9.4	65.659	86.462	74.763	63.165	85.620	72.212
	**√**	**√**	ResNet50	7.2	67.413 (+7.5)	87.230	76.087	65.244 (+4.8)	86.824	74.499
**√**	**√**		ResNet50	9.3	65.278 (+5.4)	86.302	73.891	63.627 (+3.0)	85.737	73.235
**√**	**√**	**√**	ResNet50	7.1	**68.501 (+8.6)**	**87.433 (+1.8)**	**77.793 (+9.0)**	**65.650 (+5.0)**	**87.081 (+2.2)**	**75.643 (+6.5)**

All models employ focal loss during the training period with γ=0.25 and α=2.0 .

FPS, number of images processed per second.

The six bold values are the accuracies of the best models. For the specific meaning of accuracies, refer to the table header. The values ​​in brackets are the added values ​​compared to the first row of **Table 3**.

##### 3.4.2.1 Effect of DCN

**Figure 11:**
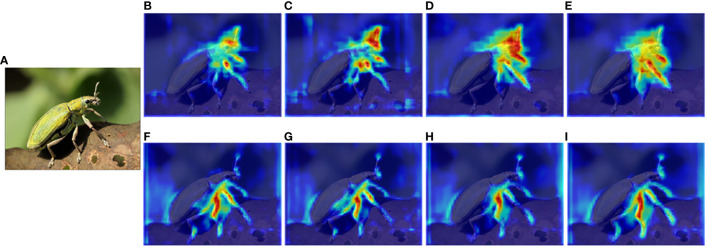
Visualization results of feature extraction after the backbone network fuses different modules. **(A)** Input, **(B)** FPN, **(C)** FPN fused with self-attention, **(D)** FPN fused with DCN, **(E)** FPN fused with DCN and self-attention (deformable attention block), **(F)** FaPN, **(G)** FaPN fused with self-attention, **(H)** FaPN fused with DCN, and **(I)** FaPN fused with DCN and self-attention (deformable attention block).

DCN learns by updating the offset, allowing the convolution kernel to align more closely with the shape and size of the object during sampling. This approach proves to be efficient for segmenting densely distributed and mimetic tea pests. Experimental results show that employing DCN enhances the accuracy of Transfiner, either integrating self-attention and FaPN or not, in both tea pest detection and segmentation tasks. Besides this, the integration of DCN refines the edge feature extraction results to detailed areas such as the insect’s antennae and legs, as shown in the feature extraction visualization outputted by the pyramid network (**Figure 11**).

##### 3.4.2.2 Effect of self-attention

As an essential component of the Transformer architecture, the module aims to extract global features from input images. As shown in **Table 3**, it can be observed that before integrating DCN, the fusion of this attention module leads to a decrease in the model’s detection and segmentation performance. However, incorporating DCN with self-attention (the deformable attention block) into the backbone results in a subtle improvement on detection and segmentation accuracy. It is noteworthy that while self-attention does not significantly improve accuracy, it enables the backbone network to focus more on detailed information such as the legs and antenna of pests, as shown in **Figure 11**. This mechanism has a significant impact on the TP-Transfiner’s ability to segment mimetic pest with slender antennae.

##### 3.4.2.3 Effect of feature-aligned pyramid network

FaPN improves the feature misalignment issue of FPN, particularly around the border area. Therefore, it assists TP-Transfiner in enhancing the feature extraction ability for pest edge, leading to more accurate segmentation of pests in mimicry. A strong comparison depicted in the feature extraction visualization (**Figure 11**) shows that when FaPN is fused (the second line), the most attended area is distributed around the legs and antennae of the pest. As for the detection and segmentation accuracy, FaPN significantly improves model performance, regardless of whether the self-attention and DCN modules are integrated (as shown in **Table 3**).

## 4 Conclusion

To address the limitations of tea pest detection and segmentation in dense and mimicry scenarios, this study develops an end-to-end framework called TP-Transfiner. The framework integrates a deformable attention block, consisting of deformable convolution and a self-attention module, to improve pest feature extraction ability. Additionally, the FPN architecture is enhanced with a more effective FaPN to address feature misalignment issues. Focal loss is introduced during the training period, and γ=2 and α=0.45 are adjusted to optimize the model’s performance. Furthermore, to solve the insufficient tea pest dataset for detection and segmentation tasks, this study conducts a TeaPestDataset including 29 categories of tea pests. Experimental results on the TeaPestDataset demonstrate that TP-Transfiner has outstanding tea pest detection and segmentation performance compared with several classic models, particularly in dense and mimicry scenarios. The model achieves state-of-the-art performance in both object detection (mAP: 67.372%) and instance segmentation (mAP: 66.123%) tasks, with the same computing resource requirements as the original model while remaining lightweight. Besides this, the deformable attention block is proven to have outstanding feature extraction ability on detailed information.

However, the proposed TP-Transfiner needs to be further improved for pest detection and segmentation in occluded scenes, and it is inefficient for the accurate detection of pests in real-time applications. Therefore, future work will focus on simplifying the model’s architecture. Additionally, this study plans to expand the variety and quantity of images in TeaPestDataset. These efforts aim to provide a more precise method for automating pest monitoring.

## Data Availability

The raw data supporting the conclusions of this article will be made available by the authors, without undue reservation.
